# 2-(1-Methyl-2-oxoindolin-3-yl­idene)malono­nitrile

**DOI:** 10.1107/S1600536813016012

**Published:** 2013-06-15

**Authors:** De-Cai Wang, Wei Tang, Peng Su, Ping-Kai Ou-Yang

**Affiliations:** a State Key Laboratory of Materials-Oriented Chemical Engineering, School of Pharmaceutical Sciences, Nanjing University of Technology, Xinmofan Road No. 5 Nanjing, Nanjing 210009, People’s Republic of China

## Abstract

The title mol­ecule, C_12_H_7_N_3_O, is almost planar, with an r.m.s. deviation of 0.026 Å. No directional interactions could be detected in the crystal.

## Related literature

For background literature, see: Demchuk *et al.* (2011 ▶). For the crystal structure of a related compound, see: Spencer *et al.* (2010) ▶.
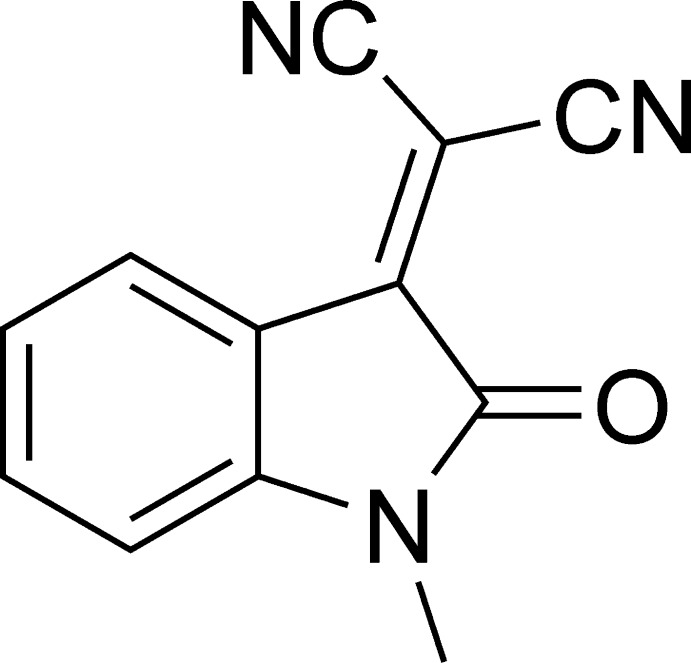


## Experimental

### 

#### Crystal data

C_12_H_7_N_3_O
*M* *_r_* = 209.21
Monoclinic, 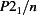

*a* = 6.9720 (14) Å
*b* = 9.929 (2) Å
*c* = 15.084 (3) Å
β = 100.25 (3)°
*V* = 1027.5 (4) Å^3^
*Z* = 4
Mo *K*α radiation
μ = 0.09 mm^−1^
*T* = 293 K
0.30 × 0.20 × 0.10 mm

#### Data collection

Enraf–Nonius CAD-4 diffractometer
Absorption correction: ψ scan (North *et al.*, 1968 ▶) *T*_min_ = 0.973, *T*_max_ = 0.991
2056 measured reflections
1896 independent reflections
1278 reflections with *I* > 2σ(*I*)
*R*_int_ = 0.081
3 standard reflections every 200 reflections intensity decay: 1%

#### Refinement

*R*[*F* ^2^ > 2σ(*F* ^2^)] = 0.057
*wR*(*F* ^2^) = 0.176
*S* = 1.00
1896 reflections
146 parameters
H-atom parameters constrained
Δρ_max_ = 0.18 e Å^−3^
Δρ_min_ = −0.19 e Å^−3^

### 

Data collection: *CAD-4 EXPRESS* (Enraf–Nonius, 1994 ▶); cell refinement: *CAD-4 EXPRESS*; data reduction: *XCAD4* (Harms & Wocadlo,1995 ▶); program(s) used to solve structure: *SHELXS97* (Sheldrick, 2008 ▶); program(s) used to refine structure: *SHELXL97* (Sheldrick, 2008 ▶); molecular graphics: *SHELXTL* (Sheldrick, 2008 ▶); software used to prepare material for publication: *PLATON* (Spek, 2009 ▶).

## Supplementary Material

Crystal structure: contains datablock(s) I, global. DOI: 10.1107/S1600536813016012/pv2634sup1.cif

Structure factors: contains datablock(s) I. DOI: 10.1107/S1600536813016012/pv2634Isup2.hkl

Supplementary material file. DOI: 10.1107/S1600536813016012/pv2634Isup3.cml

Additional supplementary materials:  crystallographic information; 3D view; checkCIF report

## Figures and Tables

**Fig. 1.**
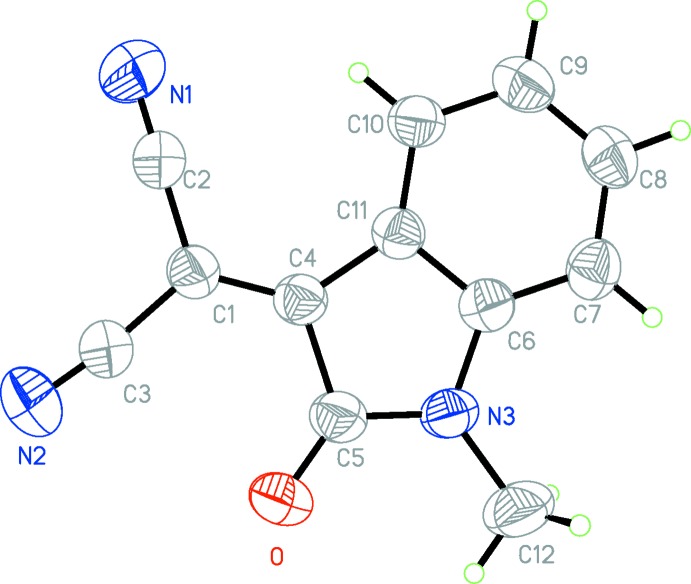
The molecular structure of the title molecule, with the atom numbering scheme. Displacement ellipsoids are drawn at the 50% probability level.

**Fig. 2.**
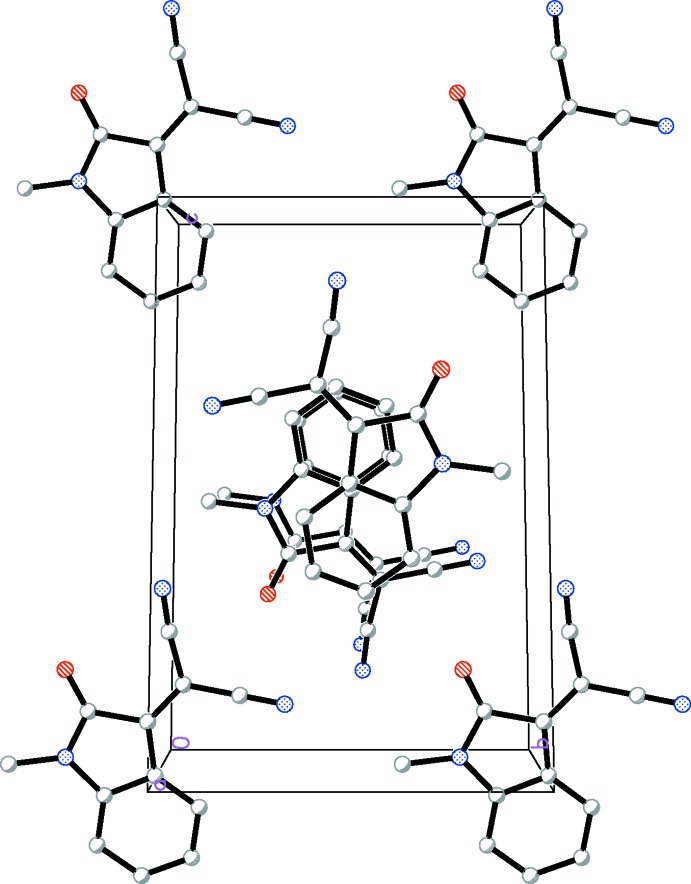
A unit cell packing diagram of the title compound.

## supplementary crystallographic information

### Comment

The title compound is an important intermediate in the synthesis of 2-(1-methyl-2-oxoindolin-3-yl)malononitrile, which in turn is a useful pharmaceutical intermediate (Demchuk *et al.*, 2011). We report herein the crystal structure of the title compound.

The bond distances and angles in the title compound (Fig. 1) agree very well with the corresponding bond distances and angles reported in a closely related compound (Spencer *et al.*, 2010). The crystal structure is devoid of any classic hydrogen bonds (Fig. 2).

### Experimental

A solution of 1-methylindoline-2,3-dione (8.1 g, 0.05 mol) in acetonitrile (20 ml) was added dropwise, while stirring, to malononitrile (6.6 g, 0.1 mol) dissolved in acetonitrile (10 ml), at room temperature. After stirring for 40 minutes, the precipitated 2-(1-methyl-2-oxoindolin-3-ylidene)malononitrile was filtered off and washed with 40 ml portions of acetonitrile, and the combined filtrates were concentrated under reduced pressure; yellow crytalline 2-(1-methyl-2-oxoindolin-3-ylidene)malononitrile, the title compound, was thus obtained (8.2 g; yield = 60%). The crystals suitable for X-ray diffraction were obtained by slow evaporation of EtOH solution.

### Refinement

All H atoms were placed geometrically at the distances of 0.93–0.97 Å for C—H and included in the refinement in riding motion approximation with *U*_iso_(H) = 1.2 or 1.5*U*_eq_ of the carrier atom.

### Crystal data

**Table:**

C_12_H_7_N_3_O	*F*(000) = 432
*M**_r_* = 209.21	*D*_x_ = 1.352 Mg m^−^^3^
Monoclinic, *P*2_1_/*n*	Mo *K*α radiation, λ = 0.71073 Å
Hall symbol: -P 2yn	Cell parameters from 25 reflections
*a* = 6.9720 (14) Å	θ = 9–13°
*b* = 9.929 (2) Å	µ = 0.09 mm^−^^1^
*c* = 15.084 (3) Å	*T* = 293 K
β = 100.25 (3)°	Block, yellow
*V* = 1027.5 (4) Å^3^	0.30 × 0.20 × 0.10 mm
*Z* = 4	

### Data collection

**Table:**

Enraf–Nonius CAD-4 diffractometer	1278 reflections with *I* > 2σ(*I*)
Radiation source: fine-focus sealed tube	*R*_int_ = 0.081
Graphite monochromator	θ_max_ = 25.4°, θ_min_ = 2.5°
ω/2θ scans	*h* = 0→8
Absorption correction: ψ scan (North *et al.*, 1968)	*k* = 0→11
*T*_min_ = 0.973, *T*_max_ = 0.991	*l* = −18→17
2056 measured reflections	3 standard reflections every 200 reflections
1896 independent reflections	intensity decay: 1%

### Refinement

**Table:**

Refinement on *F*^2^	Secondary atom site location: difference Fourier map
Least-squares matrix: full	Hydrogen site location: inferred from neighbouring sites
*R*[*F*^2^ > 2σ(*F*^2^)] = 0.057	H-atom parameters constrained
*wR*(*F*^2^) = 0.176	*w* = 1/[σ^2^(*F*_o_^2^) + (0.1*P*)^2^ + 0.150*P*] where *P* = (*F*_o_^2^ + 2*F*_c_^2^)/3
*S* = 1.00	(Δ/σ)_max_ < 0.001
1896 reflections	Δρ_max_ = 0.18 e Å^−^^3^
146 parameters	Δρ_min_ = −0.19 e Å^−^^3^
0 restraints	Extinction correction: *SHELXL97* (Sheldrick, 2008), Fc^*^=kFc[1+0.001xFc^2^λ^3^/sin(2θ)]^-1/4^
Primary atom site location: structure-invariant direct methods	Extinction coefficient: 0.082 (11)

### Special details

**Table:**

Geometry. All e.s.d.'s (except the e.s.d. in the dihedral angle between two l.s. planes) are estimated using the full covariance matrix. The cell e.s.d.'s are taken into account individually in the estimation of e.s.d.'s in distances, angles and torsion angles; correlations between e.s.d.'s in cell parameters are only used when they are defined by crystal symmetry. An approximate (isotropic) treatment of cell e.s.d.'s is used for estimating e.s.d.'s involving l.s. planes.
Refinement. Refinement of *F*^2^ against ALL reflections. The weighted *R*-factor *wR* and goodness of fit *S* are based on *F*^2^, conventional *R*-factors *R* are based on *F*, with *F* set to zero for negative *F*^2^. The threshold expression of *F*^2^ > σ(*F*^2^) is used only for calculating *R*-factors(gt) *etc*. and is not relevant to the choice of reflections for refinement. *R*-factors based on *F*^2^ are statistically about twice as large as those based on *F*, and *R*- factors based on ALL data will be even larger.

### Fractional atomic coordinates and isotropic or equivalent isotropic displacement parameters (Å^2^)

**Table:**

	*x*	*y*	*z*	*U*_iso_*/*U*_eq_	
O	0.0898 (3)	0.2173 (2)	0.80717 (13)	0.0706 (7)	
C1	0.0975 (4)	−0.0799 (3)	0.83219 (16)	0.0473 (7)	
N1	0.1159 (4)	−0.3343 (3)	0.86555 (18)	0.0819 (9)	
C2	0.1095 (4)	−0.2216 (3)	0.85071 (18)	0.0553 (7)	
N2	−0.0627 (5)	−0.0331 (3)	0.66758 (18)	0.0853 (9)	
C3	0.0117 (4)	−0.0467 (3)	0.74109 (18)	0.0583 (8)	
N3	0.2174 (3)	0.2233 (2)	0.95933 (14)	0.0526 (6)	
C4	0.1582 (3)	0.0124 (2)	0.89713 (16)	0.0439 (6)	
C5	0.1483 (4)	0.1621 (3)	0.87912 (17)	0.0505 (7)	
C6	0.2761 (3)	0.1273 (2)	1.02696 (16)	0.0452 (6)	
C7	0.3584 (4)	0.1494 (3)	1.11602 (17)	0.0535 (7)	
H7A	0.3832	0.2361	1.1385	0.064*	
C8	0.4027 (4)	0.0373 (3)	1.17050 (18)	0.0560 (7)	
H8A	0.4573	0.0493	1.2309	0.067*	
C9	0.3678 (4)	−0.0923 (3)	1.13745 (17)	0.0572 (7)	
H9A	0.3983	−0.1654	1.1759	0.069*	
C10	0.2876 (4)	−0.1141 (3)	1.04742 (16)	0.0492 (7)	
H10A	0.2649	−0.2010	1.0250	0.059*	
C11	0.2423 (3)	−0.0029 (2)	0.99178 (15)	0.0427 (6)	
C12	0.2311 (5)	0.3684 (3)	0.9719 (2)	0.0769 (10)	
H12A	0.1816	0.4126	0.9159	0.115*	
H12B	0.3649	0.3933	0.9917	0.115*	
H12C	0.1558	0.3951	1.0164	0.115*	

### Atomic displacement parameters (Å^2^)

**Table:**

	*U*^11^	*U*^22^	*U*^33^	*U*^12^	*U*^13^	*U*^23^
O	0.1073 (17)	0.0532 (12)	0.0506 (12)	0.0063 (10)	0.0123 (11)	0.0123 (9)
C1	0.0525 (15)	0.0477 (16)	0.0421 (14)	0.0037 (11)	0.0098 (11)	0.0010 (11)
N1	0.119 (2)	0.0507 (17)	0.0754 (19)	0.0038 (15)	0.0144 (16)	−0.0016 (14)
C2	0.0689 (18)	0.0507 (17)	0.0453 (15)	0.0021 (13)	0.0077 (12)	−0.0035 (13)
N2	0.116 (2)	0.089 (2)	0.0472 (15)	0.0060 (17)	0.0046 (14)	0.0044 (14)
C3	0.077 (2)	0.0517 (16)	0.0466 (17)	0.0008 (14)	0.0122 (14)	−0.0018 (13)
N3	0.0735 (15)	0.0372 (12)	0.0488 (13)	0.0004 (10)	0.0155 (11)	0.0023 (10)
C4	0.0474 (13)	0.0426 (14)	0.0429 (13)	0.0025 (10)	0.0112 (10)	0.0025 (11)
C5	0.0622 (16)	0.0464 (15)	0.0452 (15)	0.0013 (12)	0.0155 (12)	0.0052 (12)
C6	0.0472 (14)	0.0453 (15)	0.0451 (14)	0.0006 (11)	0.0135 (10)	0.0006 (11)
C7	0.0581 (16)	0.0550 (16)	0.0482 (15)	−0.0048 (12)	0.0118 (12)	−0.0078 (13)
C8	0.0581 (16)	0.0666 (19)	0.0415 (14)	0.0021 (13)	0.0042 (11)	−0.0007 (13)
C9	0.0619 (17)	0.0606 (18)	0.0477 (16)	0.0066 (13)	0.0060 (12)	0.0107 (13)
C10	0.0545 (15)	0.0454 (15)	0.0475 (14)	0.0045 (11)	0.0089 (11)	0.0044 (11)
C11	0.0436 (13)	0.0444 (14)	0.0408 (13)	0.0025 (10)	0.0095 (10)	0.0011 (11)
C12	0.122 (3)	0.0415 (17)	0.072 (2)	−0.0048 (16)	0.0294 (18)	−0.0016 (14)

### Geometric parameters (Å, º)

**Table:**

O—C5	1.220 (3)	C6—C11	1.402 (3)
C1—C4	1.353 (4)	C7—C8	1.386 (4)
C1—C2	1.434 (4)	C7—H7A	0.9300
C1—C3	1.436 (4)	C8—C9	1.386 (4)
N1—C2	1.140 (4)	C8—H8A	0.9300
N2—C3	1.146 (3)	C9—C10	1.390 (4)
N3—C5	1.363 (3)	C9—H9A	0.9300
N3—C6	1.403 (3)	C10—C11	1.388 (3)
N3—C12	1.455 (4)	C10—H10A	0.9300
C4—C11	1.452 (3)	C12—H12A	0.9600
C4—C5	1.510 (4)	C12—H12B	0.9600
C6—C7	1.381 (3)	C12—H12C	0.9600
			
C4—C1—C2	121.6 (2)	C8—C7—H7A	121.3
C4—C1—C3	124.1 (2)	C9—C8—C7	121.8 (3)
C2—C1—C3	114.3 (2)	C9—C8—H8A	119.1
N1—C2—C1	178.9 (3)	C7—C8—H8A	119.1
N2—C3—C1	173.3 (3)	C8—C9—C10	120.7 (3)
C5—N3—C6	110.7 (2)	C8—C9—H9A	119.7
C5—N3—C12	124.2 (2)	C10—C9—H9A	119.7
C6—N3—C12	125.1 (2)	C11—C10—C9	118.4 (3)
C1—C4—C11	131.3 (2)	C11—C10—H10A	120.8
C1—C4—C5	122.5 (2)	C9—C10—H10A	120.8
C11—C4—C5	106.1 (2)	C10—C11—C6	120.0 (2)
O—C5—N3	126.8 (3)	C10—C11—C4	133.4 (2)
O—C5—C4	126.9 (2)	C6—C11—C4	106.7 (2)
N3—C5—C4	106.3 (2)	N3—C12—H12A	109.5
C7—C6—C11	121.9 (2)	N3—C12—H12B	109.5
C7—C6—N3	128.0 (2)	H12A—C12—H12B	109.5
C11—C6—N3	110.1 (2)	N3—C12—H12C	109.5
C6—C7—C8	117.3 (3)	H12A—C12—H12C	109.5
C6—C7—H7A	121.3	H12B—C12—H12C	109.5
			
C2—C1—C4—C11	0.1 (4)	C11—C6—C7—C8	−1.5 (4)
C3—C1—C4—C11	178.2 (2)	N3—C6—C7—C8	179.4 (2)
C2—C1—C4—C5	179.6 (2)	C6—C7—C8—C9	0.5 (4)
C3—C1—C4—C5	−2.3 (4)	C7—C8—C9—C10	0.4 (4)
C6—N3—C5—O	−178.5 (3)	C8—C9—C10—C11	−0.4 (4)
C12—N3—C5—O	0.5 (4)	C9—C10—C11—C6	−0.5 (4)
C6—N3—C5—C4	1.6 (3)	C9—C10—C11—C4	−179.6 (2)
C12—N3—C5—C4	−179.4 (2)	C7—C6—C11—C10	1.6 (4)
C1—C4—C5—O	−1.0 (4)	N3—C6—C11—C10	−179.3 (2)
C11—C4—C5—O	178.6 (2)	C7—C6—C11—C4	−179.1 (2)
C1—C4—C5—N3	178.9 (2)	N3—C6—C11—C4	0.0 (3)
C11—C4—C5—N3	−1.5 (3)	C1—C4—C11—C10	−0.4 (5)
C5—N3—C6—C7	178.0 (2)	C5—C4—C11—C10	−179.9 (3)
C12—N3—C6—C7	−0.9 (4)	C1—C4—C11—C6	−179.6 (2)
C5—N3—C6—C11	−1.1 (3)	C5—C4—C11—C6	0.9 (3)
C12—N3—C6—C11	179.9 (2)		
